# Personalized training as a promoter for physical activity in people with depressive disorder—a randomized controlled trial in Germany

**DOI:** 10.3389/fpsyt.2023.1158705

**Published:** 2023-06-29

**Authors:** Katriona Keller-Varady, Sven Haufe, Elisabeth Schieffer, Arno Kerling, Uwe Tegtbur, Kai G. Kahl

**Affiliations:** ^1^Department of Rehabilitation and Sports Medicine, Hannover Medical School, Hannover, Germany; ^2^Department of Psychiatry, Social Psychiatry and Psychotherapy, Hannover Medical School, Hannover, Germany

**Keywords:** depression, exercise, physical activity, motivation, motivational interviewing, sport

## Abstract

**Introduction:**

Adopting an active lifestyle is an important goal, but can be difficult to achieve for people with depressive disorders. Current guidelines recommend the integration of physical activity in the multimodal treatment of depressive disorders. However, the possibilities to provide individual support for physical activities are frequently limited. The aim of our study was to examine how physical activity can be increased in a real-world setting by combining physical training and psychological interventions.

**Materials and methods:**

In this randomized-controlled interventional study, 31 outpatients diagnosed with moderate to severe depression were recruited from the region of Hannover. The intervention group (*n* = 16) was offered six weekly individual sessions lasting between 60 and 90  min with a sports scientist, including Motivational Interviewing and accompanied exercise activities. The control group (*n* = 15) received a written booklet with information on steps toward becoming more active. Moderate-to-vigorous physical activity (MVPA) as the primary outcome was analyzed using activity sensors before and after the 6-week intervention, and 3  months subsequently. Secondary outcomes included the Six-Minute Walk Test (6MWT), Sit-to-Stand test (STS), and mental health assessed with self-rating questionnaires.

**Results:**

In the intervention group, MVPA increased significantly between baseline and the first follow-up and remained at an increased level at the second follow-up in comparison to decreased levels in the control group (difference of 15.5  min/day between groups over time, SE = 6.2  min/day, 95%-CI[2.7, 28.3], *p* = 0.020). The increased activity level was associated with markers of increased fitness (6MWT and STS) in the intervention group. Both groups showed comparable improvements in depressive symptoms, while the number of patients receiving antidepressants increased in the control group and decreased in the intervention group. Two patients dropped out of the intervention group during the trial.

**Conclusion:**

The intervention proved to be a feasible and effective aid to promote a physically active lifestyle for patients diagnosed with depression. Furthermore, the higher level of physical activity was maintained for the follow-up period. Given the success of the approach evaluated in this project, individual support for physical activity should be investigated in larger sample sizes and potentially be considered in the multimodal treatment of depression.

**Clinical trial registration:**

[https://clinicaltrials.gov/], identifier [DRKS00023257].

## 1. Introduction

Achieving a change in exercise habits is an important goal in patient care for depression, but is frequently difficult to implement in practice. Loss of interest, energy, self-confidence and decisiveness are important determinants of major depressive disorder and impede patients in following an active lifestyle. Indeed, studies and meta-analyses demonstrate that people with depression tend toward sedentary lifestyles with decreased physical activity compared with healthy controls (1–4). There is also evidence for an inverse relationship between physical activity and depression [e.g., (5–8)] and an increased risk for inactivity-induced physical comorbidities [e.g., (9)]. However, the promotion of physical activity for patients suffering from depression harbors significant therapeutic potential (10). Starting an active lifestyle can lead to a multitude of positive effects through various biological or psychosocial pathways [e.g., (11)], but there is currently no established or accepted method to increase activity in already depressed and inactive people. In previous studies, we have analyzed the effects of exercise interventions on adipose tissue, muscle mass, cardiorespiratory fitness, metabolic syndrome and brain-derived neurotrophic factor in inpatients with major depressive disorder (12–15) and the effects of physical activity on the severity of depression and anxiety in company employees (16). Building on these findings, the present study evaluates how to increase physical activity in “real-world” settings in outpatients with depressive disorders.

Depressed mood and stress represent the main barriers to physical activity, as do poor levels of social support (17). Lack of time, physical illness, and poor health are also stated as limiting factors (18). Furthermore, a higher body mass index and a lower self-efficacy are associated with lower participation in physical activity (19). Professional assistance in setting goals, overcoming barriers and maintaining motivation (17), and the inclusion of health care professionals qualified in exercise prescription (20) are recommended strategies to assist patients with depressive disorder. The importance of increasing autonomous motivation (21) and self-efficacy (22) is recognized and Motivational Interviewing techniques can support behavioral change on the basis of patients’ own decisions (23, 24). In previous studies, personal or telephone contact provided an opportunity for patients to speak about their goals and any potential reservations regarding exercise (25), and to explain the effects of physical activity (26). Further promising approaches identified in previous projects include personal coaching with individualized training schedules (27), a combination of supervised exercise with recommendations for home-based exercise (28), and a hybrid approach with telehealth options, web-based modules and smart technology [e.g., (29–31)]. In contrast to many previous approaches, our study neither focuses on group exercise [e.g., (32)] nor measures efficiency in reducing weight or symptom severity [e.g., (33)], but rather measures change in physical activity as the primary outcome. On the basis of previous work, we designed an intervention based on a combination of the supporting factors outlined above and techniques such as Motivational Interviewing, flanked by a new component of “accompanied activity.” This involved the discussion of barriers such as those mentioned above, and limiting factors in personal conversations with the study participants.

The aim of this study was to examine how to promote a long-term increase in self-selected physical activity in the patients’ daily life using this combination of physical training and psychological methods. We hypothesized a significantly greater increase in moderate-to-vigorous physical activity (MVPA) as measured with activity trackers when using this combined approach in the intervention group in comparison with the control group. The main focus was on the long-term effects of our intervention as assessed at the second follow-up visit, 3 months after the end of the intervention.

## 2. Materials and methods

### 2.1. Participants

**Figure 1:**
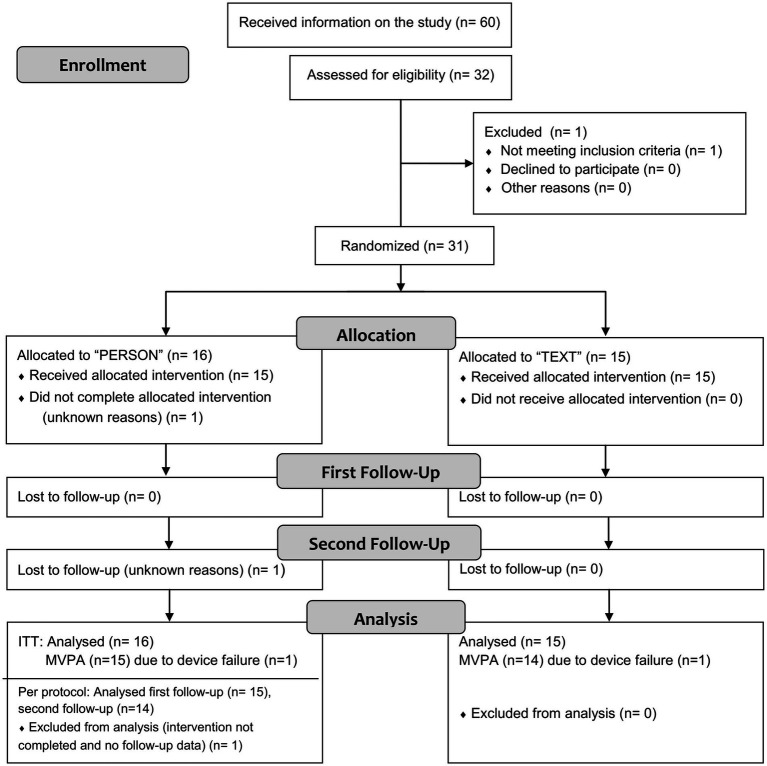
Flowchart of the numbers of participants and dropouts (for details on excluded and missing values, see paragraph “Statistical analysis” and Table 3. MVPA, moderate-to-vigorous physical activity).

We advertised for potential participants in Hannover (Lower Saxony, Germany) between November 2020 and February 2022. Initial details on the trial were distributed via posts on selected internet platforms (homepage of the “Patienten Universität an der Medizinischen Hochschule Hannover” and Hannover Medical School intranet) and on Hannover Medical School’s social media channel. Handouts were made available by health care professionals in outpatient clinics (Wahrendorff clinical center and Hannover Medical School) and medical practices (in a radius of approximately 5 km of the study site). This resulted in 60 potential participants who were subsequently informed about the study in detail (see Figure 1). The inclusion criteria for patients were: a diagnosis of depression (F32, F33) according to ICD-10 (34), age between 18 and 60 years, and place of residence in the region of Hannover. Exclusion criteria were substance abuse, suicidal tendencies, lack of ability to understand information or give informed consent, pregnancy, lactation, or contraindications for unsupervised physical activity or tests. Written informed consent was obtained from all participants. The study was approved by the local ethics committee at the Hannover Medical School (NR8924_BO_S_2020), registered at “Deutsches Register Klinischer Studien”/WHO International Clinical Trials Registry Platform (DRKS00023257) and performed in accordance with the Declaration of Helsinki. Recruitment was stopped once the required sample size was reached.

Patients were randomly assigned to either the intervention group (*n* = 16) or the control group (*n* = 15). All participants continued with their usual medications. Changes in medication were not part of the study procedure, but were monitored at each study visit. Participants changed their medication in consultation with their therapists who were not part of the study team. Twenty-four patients were taking medication; of these 18 were taking antidepressants and six were taking only cardiac or thyroid dysfunction medication. Seven patients were not taking any medication at baseline. The baseline characteristics of the groups are reported in Table 1.

**Table 1:** Baseline characteristics of participants as mean ± standard deviation or absolute number in the two groups (intervention group, personalized training with Motivational Interviewing and accompanied activity, “person”; control group, written information, “text”).

	Descriptive statistics	
Baseline characteristic	Intervention group (person)	Control group (text)
Size of group (n)	16	15
Age (y)	41.4 ± 10.6	41.1 ± 12.5
Women/men (n)	14/2	13/2
Body weight (kg)	83.2 ± 19.9	74.1 ± 13.4
BMI (kg/m^2^)	28.8 ± 6.5	25.8 ± 4.4
MADRS (points)	21.5 ± 6.1	22.9 ± 5.6
Employed yes/no (n)	7/9	7/8
Psychotherapy yes/no (n)	11/5	13/2
Antidepressants yes/no (n)	12/4	6/9

Significant group differences occurred only in the number of participants under treatment with antidepressants.

y, years; kg, kilogram; m, meters; %, percent; n, number of participants with specific characteristic; BMI, body mass index; MADRS, Montgomery-Åsberg Depression Scale.

### 2.2. Interventions

Patients in the intervention group participated in six individual sessions (one session per week) lasting between 60 and 90 min with a sports scientist, comprising Motivational Interviewing and accompanied physical activity. Sessions included theoretical and practical parts and were standardized with the help of pre-formulated lists of questions and exercises, as well as work sheets and written information. The practical part consisted of a multi-faceted exercise program under the guidance of an experienced sports therapist with training in Motivational Interviewing. The participants had the possibility to try out endurance and strength training, complementary exercises addressing flexibility, coordination, relaxation, and individually requested sports. Participants could individually select a preferred activity based on the guided testing or previous positive experiences. For the theoretical part, the participants’ past exercise experiences and current status were evaluated; goals were defined, analyzed and divided into small tasks. Discrepancies between the current and desired physical activity status were identified. Together with the patient, the sports therapist discussed the advantages and disadvantages of increased sports activity, examined factors which might promote or complicate exercise, and formulated a daily and weekly plan. General information on healthy eating and the effects of sports and exercise on health was provided. The results from the baseline assessments were explained and used to tailor the activities to the patient’s individual health and fitness status. The overall goal was to meet general activity recommendations (35) and to find individual activities that the participants were interested in continuing on their own. The sports therapist was able to act as a sports companion and join the participant’s selected activity, e.g., sports group, fitness center visit or walking tour at home (accompanied activity). The stepwise development of activities has parallels with behavioral activation strategies (36–38), regarding physical activity as a form of healthy behavior with the potential for positive experiences. Following previous studies (39, 40), the intervention is based on principles of self-determination theory (41) and uses Motivational Interviewing techniques (42, 43) (for a detailed description of the contents of the intervention, see Table 2).

**Table 2:** Contents of the intervention sessions targeted to individual needs.

Week	Theoretical part	Practical part
1	Oral information: explaining the results of visit 1 and deducing special therapeutic needs with regard to pre-existing diseases Written information: general recommendations for activity Motivational Interviewing: rating of importance of change and rating of confidence (evaluation of readiness for change), expectations, individual starting point Work sheet: “advantages and disadvantages of activity increase” Time for individual needs	Accompanied activity/guided exercise program: active breaks Activity recommendation: interrupt sitting time with an active break Time for individual activity plans
2	Oral information: effects of endurance training Written information: endurance training Motivational Interviewing: reflecting the experiences of last week, reasons for change, supporting factors, inducing “change talk” Work sheet: “what helps others, what helps me” Time for individual needs	Accompanied activity/guided exercise program: endurance activity, e.g., walking, biking Activity recommendation: repetition of endurance activity or a second endurance activity or an individual self-selected activity Time for individual activity plans
3	Oral information: effects of strength training Written information: strength training Motivational Interviewing: reflecting the experiences of last week Problem solving strategies: barriers and worries Work sheet: “barriers and ideas for solutions” time for individual needs	Accompanied activity/guided exercise program: strength training, e.g., exercises in a fitness center or at home with resistance bands Activity recommendation: repetition of strength training activity or a second strength training activity or an individual self-selected activity Time for individual activity plans
4	Oral information: possibilities of flexibility, coordination, relaxation exercises Written information: flexibility, coordination, relaxation exercises and “possibilities of support” Motivational Interviewing: reflecting the experiences of last week, talking about possible activities (list of sports activities) and the first step, inducing “change talk” Goal setting: defining an individual goal and dividing it into small tasks Work sheet: “step by step” Time for individual needs	Accompanied activity/guided exercise program: flexibility, coordination, relaxation exercises Activity recommendation: repetition of the guided activities or an individual self-selected activity Time for individual activity plans
5	Oral information: doses of activity and recovery Motivational Interviewing: reflecting the experiences of last week, talking about activity slots in a typical day or week, inducing “change talk” Work sheet: daily schedule, weekly schedule Time for individual needs	Accompanied activity: individual self-selected activity Activity recommendation: endurance activity and activity with exercises (strength, flexibility, coordination and relaxation) and individual self-selected activity Time for individual activity plans
6	Oral information: long-term training schedule Motivational Interviewing: reflecting the experiences of the intervention, rating of importance of change and rating of confidence, talking about future plans, inducing “change talk” Work sheet: “looking back and looking ahead” Time for individual needs	Accompanied activity: - Activity recommendation: endurance activity and activity with exercises (strength, flexibility, coordination and relaxation) and individual self-selected activity Time for individual activity plans

Patients in the control group received written information after the results of the baseline assessments had been explained. It contained detailed information on safety, activity recommendations of World Health Organization (WHO) (35) and American College of Sports Medicine (ACSM) (44), training methods, sports disciplines, organization, sports equipment, tips for motivation, information on healthy eating and an exercise log. The control setting did not provide the option of any personal contact in the intervention period, or any individualization of the contents or accompanied activity; all other assessments were identical to those in the intervention group.

### 2.3. Assessment of physical activity

**Figure 2:**
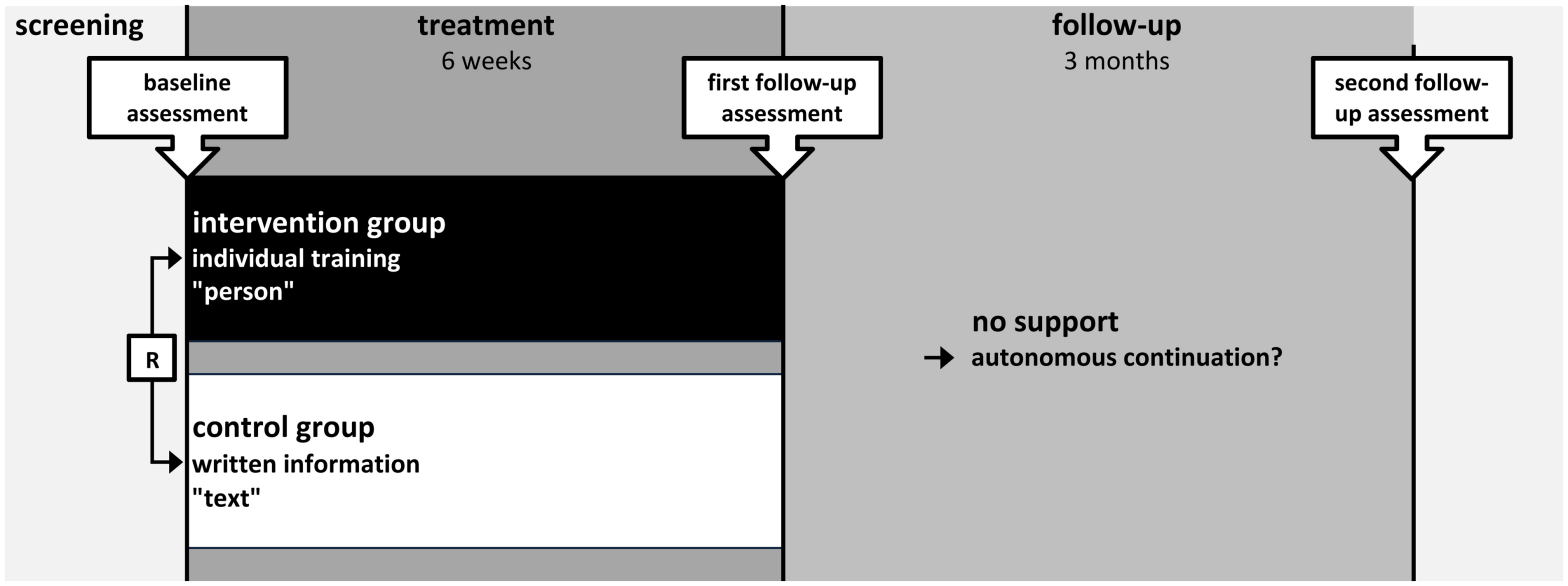
Timeline of the assessments and periods (R: randomization).

As shown in Figure 2, data was collected at baseline (visit 1), directly after the intervention period (visit 2, first follow-up), and after a three-month period following the intervention, during which no training or assistance was offered (visit 3, second follow-up).

Moderate-to-vigorous physical activity (MVPA) in min/day was defined as the primary outcome and was assessed with waist-worn activity sensors (GT9X Link, ActiGraph, Pensacola, Florida, United States). These are medical devices that measure acceleration in the vertical, medio-lateral and antero-posterior axis (45, 46). The activity was measured over 7 days according to a previously defined protocol. Data was processed and analyzed with ActiLife 6 (ActiGraph, Pensacola, Florida, United States) following the recommendations of a systematic review of previous studies (47). In order to detect measurement errors, participants were asked to record non-wear times, activities in water, or activities with high energy expenditure but low acceleration (e.g., stationary bicycle), as well as biking to recognize the use of e-bikes. In the results section we report the measured values without manual correction to minimize evaluator influence or bias.

In addition, the self-rating “Freiburger Questionnaire on Physical Activity” was used (48) to confirm measurements. This also allows the separate analyses of sports activities.

### 2.4. Secondary parameters of physical health

Changes in fitness were assessed using simple tests of everyday activities: Sit-to-Stand Test (STS) (49) and Six-Minute Walk Test (6MWT) (50) with standardized test protocols.

### 2.5. Secondary parameters of mental health

Self-rating questionnaires were used: World Health Organization Quality of Life-Bref (WHOQOL-Bref) (51), Beck Depression Inventory (BDI II) (52), and “Barriers and barrier management in physical exercise” (53). The Montgomery-Åsberg Depression Scale (MADRS) (54, 55) was used as an expert rating in order to measure the severity of depressive symptoms.

### 2.6. Power analysis

The required sample size for the primary outcome (MVPA) was 15 per group according to the *a priori* power analysis with SAS/STAT Software (SAS Institute Inc., Cary, United States) with the following assumptions: adjusted type I significance level of α = 0.05, power of 1 – β = 0.8, MVPA-differences between groups at visit 3 of 27 min/day, standard deviation 25 min/day. Expected values were based on literature research (10).

### 2.7. Statistical analysis

Statistical analysis was performed with IBM SPSS Statistics (Version 22, International Business Machines Corporation, Armonk, New York, United States). After testing for normal distribution (Kolmogorov–Smirnov test, Shapiro Wilk test and histogram), the groups were compared either by analysis of variance (ANOVA) or the Mann–Whitney-*U*-test. The effect of time and time x group interactions were analyzed using the general linear model for repeated measurements with three factor levels and Bonferroni-corrected post-hoc tests or the Friedman test as the appropriate nonparametric alternative (56). We focused on the results of the parametric tests because they are described as robust against violations of the normal distribution (57). As a measure of effect size, partial η^2^ is reported (η^2^ ≥ 0.01: small effect; ≥0.06: medium effect; ≥0.14: large effect). Individual changes over time were compared between groups with analysis of covariance (ANCOVA) adjusted for baseline values. The difference between the means of both groups is reported as MD. The level of significance was *α* = 0.05. In the results section, we report the results of the Intention-To-Treat (ITT) analysis to avoid an overestimation of effects (58). Missing values due to dropout were imputed using the baseline observation carried-forward method. Single missing values, e.g., as a result of device failure, were not imputed. The Spearman-Rho and Pearson correlation coefficients were calculated for the analyses of relationships between parameters. Covariates are stated in the results section.

## 3. Results

### 3.1. Participants

Figure 1 shows a flowchart of the number of participants and dropouts. Twenty-six women and four men with a mean age of 41 ± 12 years and a mean body mass index (BMI) of 28 ± 6 kg/m^2^ completed the study. Eighteen participants (58%) were overweight (BMI ≥ 25 kg/m^2^), 11 Participants (35.5%) were obese (BMI ≥ 30 kg/m^2^). The baseline comparisons between groups and additional baseline characteristics are given in Table 1. The differences between the groups did not reach statistical significance except for the treatment with antidepressants.

### 3.2. Dropouts

One patient dropped out due to missed appointments (intervention and first follow-up) for unknown reasons. Another patient in the intervention group was lost to follow-up (second follow-up, visit 3) for unknown reasons. The dropout rate over the study period, from baseline to the second follow-up, was 6%.

### 3.3. Intervention

Attendance, an indicator for compliance, was 96% in the intervention group. Two appointments were canceled because of the participants’ workload, one was forgotten, and one did not take place due to dropout. Appointments were postponed in cases of scheduling difficulties or illness. In general, 73% of the appointments were held face-to-face, 27% were conducted via telephone, e-mail or video conference due to infection containment measures in the pandemic or to promote independent activity (six sessions).

The entire intervention period was impacted by the Covid-19 pandemic, for example lockdown of fitness centers. All activities had to be conducted in accordance with the varying official and individual safety precautions. Participants rated the influence of the pandemic situation on their physical activity with 7 out of 10 points (0: no influence and 10: significant influence) with 57% reporting reduced physical activity. In addition, patients rated the influence of the pandemic on their mental health with 7 out of 10 points (worsening of symptoms reported by 75%). The pandemic situation may also have influenced the choice of sports disciplines. Intervention records show that the main disciplines were walking and jogging, although 88% of the patients combined two activities. No team sports were chosen and a preference for individual disciplines was observed, e.g., swimming, yoga and fitness training. Ninety-four percent of the participants were active on their own; 75% joined a local sports group. Patients rated the intervention with 9 out of 10 points (0: not helpful; 10: very helpful). The items “personal contact” (9.6 points), “free of charge” (9.7 points) and “accompanied activity” (9.5 points) were rated as the most helpful aspects. When asked whether their physical activity was impacted by the intervention, 75% of the intervention group answered with “yes,” while only 13% of the control group reported changes in activity. 81% of interventions resulted in the completion of a sports program and increase in physical activity, and were rated as successful by the sport therapist.

No serious adverse events occurred, but 14 adverse events were reported. Only two were caused by physical activity (twisted knee and heel spur) and occurred in the control group.

### 3.4. Physical activity

**Figure 3:**
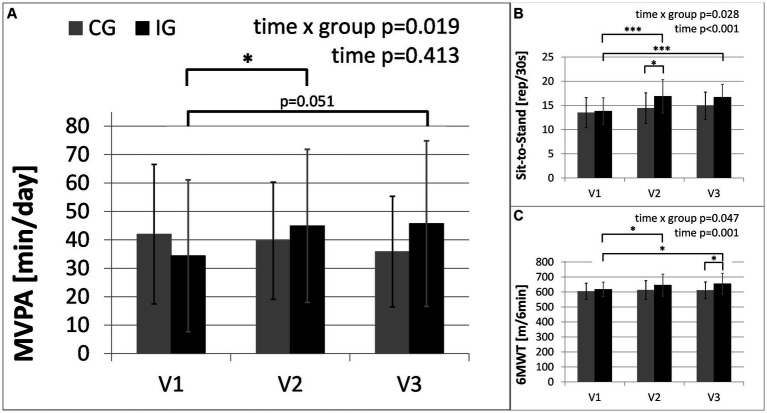
Main outcome of physical activity and secondary outcomes of physical health: **(A)** moderate-to-vigorous physical activity (MVPA) in minutes per day, assessed with activity sensors (CG, *n* = 14; IG, *n* = 15, due to failed measurements); **(B)** results of Six-Minute Walk Test (6MWT); **(C)** results of Sit-to-Stand Test. The intervention group (IG) is compared to the control group (CG) over the study period from baseline (V1) to the first follow-up visit (V2) directly after the intervention and the second follow-up (V3), 3 months after the end of the intervention (*: significant time effect/group difference *p* < 0.05; ***p < 0.001).

Results of the ANOVA with repeated measurements for all three time points revealed a significant interaction between group and time [*F*(2, 54) = 4.283, *p* = 0.019, partial η^2^ = 0.137] as shown in Figure 3. Interactions remain significant after correction with the covariates baseline symptom severity or medication dose (*p* = 0.029 and *p* = 0.035, respectively).

Between baseline and the second follow-up, MVPA increased by 10.4 min/day (SE = 4.3 min/day) in the intervention group, but decreased by about 5.1 min/day (SE = 4.5 min/day) in the control group (MD = 15.5 min/day, 95%-CI[2.7, 28.3], *p* = 0.020). Between baseline and the first follow-up, MVPA increased by 9.8 min/day (SE = 3.4 min/day) in the intervention group, and decreased by 1.5 min/day (SE = 3.5 min/day) in the control group (MD = 11.3 min/day, 95%-CI[1.1, 21.4], *p* = 0.031). Between the first and second follow-up, MVPA increased by 0.6 min/day (SE = 4.8 min/day) in the intervention group, and decreased further by 3.6 min/day (SE = 4.9 min/day) in the control group (MD = 4.2 min/day, 95%-CI[−10.0, 18.4], *p* = 0.247).

After adjusting for baseline values, MVPA at the second follow-up was significantly greater in the intervention group compared with the control group [*F*(1, 26) = 6.157, *p* = 0.020, partial η^2^ = 0.191].

The statistical results were confirmed when applying nonparametric tests (for variables with missing normal distribution) and analyzing the according-to-protocol population.

The results of the “Freiburger Questionnaire on Physical Activity” showed that sports activity significantly increased by 2.7 kcal/kg/week (SE = 1.2 kcal/kg/week) in the intervention group, but decreased by 1.6 kcal/kg/week (SE = 1.2 kcal/kg/week) in the control group between baseline and the second follow-up (MD = 4.3 kcal/kg/week, 95%-CI[0.8, 7.8], *p* = 0.019).

### 3.5. Secondary outcomes

**Figure 4:**
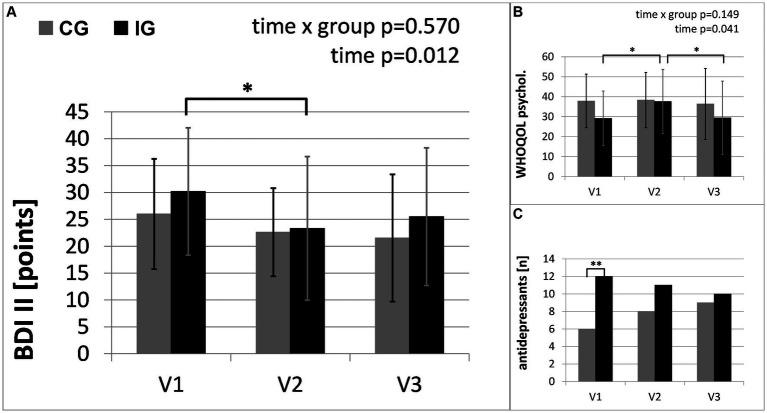
Secondary outcomes of mental health: **(A)** results of self-rating Beck Depression Inventory (BDI II); **(B)** results of World health Organization Quality of Life Questionnaire (WHOQOL-Bref); **(C)** number of participants taking antidepressants. The intervention group (IG) is compared to the control group (CG) over the study period from baseline (V1) to the first follow-up visit (V2) directly after the intervention and the second follow-up (V3), 3 months after the end of the intervention (*: significant time effect/group difference *p* < 0.05; **p < 0.01).

Table 3 provides the results of the exploratory analysis for selected secondary outcomes. Significant time x group interactions occurred in the results of the Sit-to-Stand Test (STS) and Six-Minute Walk Test (6MWT), representing the changes in physical fitness. In the intervention group STS and 6MWT increased by 21 and 6%, respectively. Both groups show significant improvements in the severity of depressive symptoms. Furthermore, Table 3 shows changes in the use of antidepressants. The changes in medication reflected a reduced number of patients taking antidepressants in the intervention group and an increased number of patients taking antidepressants in the control group (see Figure 4). No significant interactions were found between changes in depressive symptoms, physical activity and medication. In the “Barriers and barrier management in physical exercise” questionnaire, significant improvements were seen in barrier management in the intervention group (16% increase) with a significant time x group interaction for barrier management, but not for barrier severity. The management of barriers is significantly correlated with sports activity at visit 2 (*r* = 0.597, *p* = 0.001) and visit 3 (*r* = 0.489, *p* = 0.007). The perception of barriers to physical activity at baseline (*r* = −0.412, *p* = 0.024) and the results of 6MWT at baseline (*r* = −0.364, *p* = 0.048) correlated significantly with age, while all other parameters did not show significant correlations with age. No significant correlations with BMI or symptom severity were found.

**Table 3:** Secondary outcomes as mean ± standard deviation in the intervention group and control group before (visit 1), directly after the intervention (visit 2) and at the second follow-up 3 months after the end of the intervention (visit 3).

	Intervention group (person)				Control group (text)				
	n	Visit 1	Visit 2	Visit 3	n	Visit 1	Visit 2	Visit 3	Time × group interaction
PA FQ (kcal/kg/week)	16	25 ± 20	34 ± 19*	28 ± 18**	15	25 ± 15	22 ± 13	25 ± 14	*F*(2, 58) = 2.454, *p* = 0.095, partial η^2^ = 0.078
SA FQ (kcal/kg/week)	16	1 ± 3	9 ± 10	6 ± 6**	15	6 ± 12	4 ± 6	**2 ± 3**	*F*(2, 58) = 5.060, ***p*** = **0.014**, partial η^2^ = 0.149
STS (rep/30 s)	16	14 ± 3	**17 ± 4**	17 ± 3***	14	14 ± 3	**14 ± 3**	15 ± 3	*F*(2, 56) = 3.862, ***p*** = **0.028**, partial η^2^ = 0.120
6MWT (m/6 min)	16	618 ± 48	646 ± 73	655 ± 69**	14	604 ± 54	613 ± 62	**611 ± 56**	*F*(2, 56) = 3.243, ***p*** = **0.047**, partial η^2^ = 0.104
BDI II (points)	16	30 ± 12	23 ± 13	26 ± 13*	15	26 ± 10	23 ± 8	22 ± 12	*F*(2, 58) = 0.568, *p* = 0.570, partial η^2^ = 0.019
MADRS (points)	15	22 ± 6	17 ± 8	14 ± 10*	15	23 ± 6	17 ± 7	13 ± 8***	*F*(2, 56) = 0.308, *p* = 0.736, partial η^2^ = 0.011
PA-barriers (points)	16	33 ± 7	32 ± 6	32 ± 6	15	34 ± 5	34 ± 5	32 ± 6	*F*(2,58) = 0.467, *p* = 0.591, partial η^2^ = 0.016
Management (points)	16	32 ± 5	**38 ± 8**	37 ± 6*	14	34 ± 6	**31 ± 9**	32 ± 8	*F*(2, 56) = 4.753, ***p*** = **0.012**, partial η^2^ = 0.145
WHOQOL psychol. (points)	16	29 ± 14	38 ± 16	29 ± 18*	15	38 ± 13	38 ± 14	36 ± 18	*F*(2, 58) = 1.969, *p* = 0.149, partial η^2^ = 0.064
WHOQOL physical (points)	16	47 ± 16	54 ± 17	51 ± 14*	15	56 ± 14	55 ± 14	54 ± 17	*F*(2, 58) = 2.759, *p* = 0.072, partial η^2^ = 0.087
Antidepressants (n)	16	**12**	11	10	15	**6**	8	9	–

The presented results were confirmed by non-parametric analyses for not normally distributed parameters. *p*-values are not corrected for multiple testing (MVPA, moderate-to-vigorous physical activity; PA FQ, Physical Activity Freiburger Questionnaire; SA FQ, Sports Activity Freiburger Questionnaire; STS, Sit-to-Stand Test; 6MWT, Six-Minute Walk Test; BDI II, Beck Depression Inventory II; MADRS, Montgomery-Åsberg depression rating scale; PA-Barriers, barriers to physical exercise; WHO QOL, World Health Organization Quality of Life. *Time effect in repeated measurements ANOVA *p* < 0.05; **p < 0.01; ***p < 0,001; bold: difference between groups in *t*-test *p* < 0.05).

## 4. Discussion

This study demonstrates a feasible and effective way to promote a long-term increase in self-selected physical activity in a real life setting for patients with depression, combining both physical training and psychological methods.

### 4.1. Effectivity for the increase in moderate-to-vigorous physical activity

The intervention group showed a greater increase in moderate-to-vigorous physical activity than the control group. Since the control group was offered the same information via a written booklet and the same assessment visits, this difference can be attributed to the personal contact in combination with accompanied physical activity and an individualized, flexible approach to determine the best way to start activity in the Motivational Interviewing conversations. In addition to measured physical activity increases and the supporting results from the questionnaires, nearly 75% of the participants of the intervention group reported an increase in physical activity due to the intervention. Furthermore, we were able to show that parameters of physical health improved and medication with antidepressants was reduced in the intervention group in the course of the study period. Both groups showed decreased symptoms of depression. This was accompanied by a reduced use of antidepressants in the intervention group and an increased use in the control group. The periodic appointments for study assessments, together with the corresponding social contact and improved connection with the healthcare institution may have contributed to the improved mood in the control group.

In a study by Chalder et al., “physical activity facilitators” offered interviews including Motivational Interviewing techniques primarily via telephone (39, 40). This did not result in any significant reductions in depressive symptoms or medication dosage, but participants stated that they felt well and benefited from the intervention, although they would have wished for more active help. In our study, the increase in activity was smaller than expected, but baseline values were comparable to other measurements with similar activity sensors (10). Possible causes could be linked to the pandemic containment measures and fear of infection. In line with other studies (59), more than 50% of our participants reported reduced physical activity during the COVID-19 pandemic. The chosen activities, which showed a preference for activities which could be done alone and outside, and the rating of the influence of COVID-19 are both relevant in this context. However, despite the limited increase, current research has shown that even small gains in physical activity are effective in improving mental health (60).

### 4.2. Sustainability of effects

In contrast with the INSHAPE program which involved weekly contact with “health mentors” and a free sports program for 6 months leading to increased self-reported activity (61), participants in our project were not supported after the initial six weekly sessions. Nevertheless, the intervention group still showed an increased level of physical and sports activity at the second follow-up 3 months after the end of the intervention (see Figure 3). The parameters of physical health (STS and 6MWT) show similar improvements over time. In contrast, the secondary outcomes of mental health show different changes. Following an initial improvement in mental health during the intervention, they show a slight decline in the follow-up period when there was no contact with the study personnel. Thus, regular appointments and personal contact and support may be critical factors in improving patients’ mental health. Continuous follow-up appointments at a higher frequency than 3-month intervals or alternative methods to increase social support may lead to improved outcomes.

### 4.3. Feasibility and safety

Adverse events caused by physical activity were reported only in the control group and may be explained by too little knowledge of training methods and too much training load without an appropriate habituation process. On the basis of these data, the intervention was deemed safe for the participants. The intervention was also deemed feasible: Symptoms of depression, the COVID-19 lockdown, family difficulties, professional activities, orthopedic problems and pain were reported as the main barriers, but the small dropout rate of 6%, the high attendance rate of 96% and the positive ratings of the components in the intervention group suggest a good feasibility and the effectiveness of motivational strategies for the participants. The dropout rate was smaller than expected and significantly below that reported in a meta-analysis (20) for people with depression (18%). The study was feasible even under pandemic conditions in which there were strict restrictions for sports activity due to the individual training mode chosen for the intervention, in contrast with other study concepts involving group exercise [e.g., (25)].

### 4.4. Successful features of the intervention

Feedback from the patients in the intervention group showed that it was helpful that the intervention was free of charge, presented personally, and included accompanied activity. The analysis of training logs showed that structure, social connections and proximity to their place of residence were important factors. The planning and provision of short workout sequences which could be integrated in daily life and a planned structure for a 7-day period were seen as effective aids. Exploring possibilities for sport near the patient’s place of residence, contacting existing groups or finding partners for sports activity in the family or among friends (to increase social support) were also reported as helpful strategies. Beyond that, feasibility could be further improved by allowing more flexibility in making appointments, e.g., individualizing the time intervals between the appointments. Additional factors for consideration are varying requirements for individual support and the need for extra support during challenging life situations, e.g., change of residence or occupation.

### 4.5. Implications for the implementation in health care

Not all barriers to an active lifestyle are related to the patients. Garvey et al. (24) identified barriers to the prescription of exercise on the part of healthcare providers: lack of knowledge on how to prescribe exercise and establish contact with trained physical activity specialists, concerns about the patient’s ability to exercise, risk factors, such as comorbidities, or placing more stressors on their clients (24). Improvement in cooperation between the different professions is needed in addition to the establishment of routine referral methods with the possibility of individual support for physical activity. Physical exercise is recommended in the guidelines for the treatment of depression (62). Consequently, the translation of the treatment guidelines into clinical practice is the next important step. The German “Rezept für Bewegung” (translation: “prescription of activity”) by the German Olympic Sports Confederation and German Medical Association or global health initiatives such as “Exercise is Medicine®” by the American College of Sports Medicine (ACSM) already pursue this goal, but physical activity is still underused in the therapy of patients with depression (18). A more widespread application is desirable, but would need to take account of differences in country-specific health care systems and lifestyles.

### 4.6. Limitations

The present study has some limitations: Both groups experienced changes in therapy and medication during the study participation. A critical point for the statistical analysis is the small sample size. While larger effects can be detected with sufficient power, it is possible that small to medium effects would only reach statistical significance in bigger samples. Also, fewer men than women participated. Although the primary outcome was measured with activity sensors, data for secondary outcomes were collected with self-rating questionnaires and therefore may have been influenced by social desirability. The activity sensors used in the study measured acceleration, but not heart rate or effort. Therefore, the classification of activity intensity may not be sufficiently accurate. However, to address this potential limitation, we used wear time and activity logs as well as activity questionnaires and checked each automatic analysis. It should be taken into consideration that absolute values measured with varying activity sensors lack comparability between studies [e.g., (63)]. Our study procedure was furthermore impacted by the pandemic situation and its effects on physical activity (59) and the follow-up results were influenced by COVID-19 infections in two cases.

### 4.7. Perspectives

Future research should target motivational aspects of being physically active for people with mental illness and seek greater synergies between the fields of psychology and sports and training medicine. On a larger scale, projects are needed which analyze the cost–benefit ratio and test the implementation of programs supporting physical activities in the healthcare system (64). Additionally, larger samples would allow the comparison of single features of the interventions and the analysis of the influence of moderating factors and predictors of response.

### 4.8. Conclusion

Personalized training with Motivational Interviewing and accompanied physical activity was a feasible and effective aid to increase moderate-to-vigorous physical activity and improve physical performance as measured with Sit-to-Stand Test and Six-Minute Walk Test in people with depression. The combination of physical training and psychological methods focusing on individual activity preferences, needs, and possibilities in the everyday life of the participants resulted in a low dropout rate and improved management of barriers preventing physical activity. Participants in the intervention group showed improvements in depressive symptoms in spite of reductions in their pharmacological therapy. The integration of individual support regarding physical activity, including the successful methods evaluated in this project, should be considered for the multimodal treatment of depression.

## Data availability statement

The raw data supporting the conclusions of this article will be made available by the authors, without undue reservation.

## Ethics statement

The studies involving human participants were reviewed and approved by the Ethics Committee of Hannover Medical School. The patients/participants provided their written informed consent to participate in this study.

## Author contributions

KK-V, SH, AK, UT, and KK planned and designed the study. ES and AK recruited the participants. KK-V, ES, and KK collected the data. KK-V and SH were responsible for the analysis and interpretation of the data. KK-V wrote the first draft of the manuscript. SH, ES, AK, UT, and KK contributed to the discussion, reviewed, and edited the manuscript. All authors contributed to the article and approved the submitted version.

## Funding

This study was supported by the Robert-Enke-Stiftung (Schillerstraße 4, 30890 Barsinghausen, Germany). This publication is funded by the Deutsche Forschungsgemeinschaft (DFG) as part of the “Open Access Publikationskosten” program.

## Conflict of interest

The authors declare that the research was conducted in the absence of any commercial or financial relationships that could be construed as a potential conflict of interest.

## Publisher’s note

All claims expressed in this article are solely those of the authors and do not necessarily represent those of their affiliated organizations, or those of the publisher, the editors and the reviewers. Any product that may be evaluated in this article, or claim that may be made by its manufacturer, is not guaranteed or endorsed by the publisher.
